# Application of CT and MRI images based on an artificial intelligence algorithm for predicting lymph node metastasis in breast cancer patients: a meta-analysis

**DOI:** 10.1186/s12885-023-11638-z

**Published:** 2023-11-22

**Authors:** Cheng-Jie Liu, Lei Zhang, Yi Sun, Lei Geng, Rui Wang, Kai-Min Shi, Jin-Xin Wan

**Affiliations:** 1 Department of Information Center, Lianyungang Human Resources and Social Security Bureau, Lianyungang, 222000 JiangSu China; 2 Department of Information System, Lianyungang 149 Hospital, Lianyungang, 222000 Jiangsu China; 3 Department of Medical Imaging, The Second People’s Hospital of Lianyungang, 161 Xingfu Road, Haizhou District, Lianyungang, 222000 Jiangsu China; 4 Department of Information Center, Lianyungang Shuangcheng Information Technology Co., Ltd, Lianyungang, 222000 China

**Keywords:** Breast cancer, Lymph node Metastasis, CT, MRI, Artificial intelligence

## Abstract

**Background:**

This study aimed to comprehensively evaluate the accuracy and effect of computed tomography (CT) and magnetic resonance imaging (MRI) based on artificial intelligence (AI) algorithms for predicting lymph node metastasis in breast cancer patients.

**Methods:**

We systematically searched the PubMed, Embase and Cochrane Library databases for literature from inception to June 2023 using keywords that included ‘artificial intelligence’, ‘CT,’ ‘MRI’, ‘breast cancer’ and ‘lymph nodes’. Studies that met the inclusion criteria were screened and their data were extracted for analysis. The main outcome measures included sensitivity, specificity, positive likelihood ratio, negative likelihood ratio and area under the curve (AUC).

**Results:**

A total of 16 studies were included in the final meta-analysis, covering 4,764 breast cancer patients. Among them, 11 studies used the manual algorithm MRI to calculate breast cancer risk, which had a sensitivity of 0.85 (95% confidence interval [CI] 0.79–0.90; *p* < 0.001; I^2^ = 75.3%), specificity of 0.81 (95% CI 0.66–0.83; *p* < 0.001; I^2^ = 0%), a positive likelihood ratio of 4.6 (95% CI 4.0–4.8), a negative likelihood ratio of 0.18 (95% CI 0.13–0.26) and a diagnostic odds ratio of 25 (95% CI 17–38). Five studies used manual algorithm CT to calculate breast cancer risk, which had a sensitivity of 0.88 (95% CI 0.79–0.94; *p* < 0.001; I^2^ = 87.0%), specificity of 0.80 (95% CI 0.69–0.88; *p* < 0.001; I^2^ = 91.8%), a positive likelihood ratio of 4.4 (95% CI 2.7–7.0), a negative likelihood ratio of 0.15 (95% CI 0.08–0.27) and a diagnostic odds ratio of 30 (95% CI 12–72). For MRI and CT, the AUC after study pooling was 0.85 (95% CI 0.82–0.88) and 0.91 (95% CI 0.88–0.93), respectively.

**Conclusion:**

Computed tomography and MRI images based on an AI algorithm have good diagnostic accuracy in predicting lymph node metastasis in breast cancer patients and have the potential for clinical application.

## Introduction

Breast cancer is one of the most common malignant tumours in women and its incidence continues to rise worldwide. According to the World Health Organization, breast cancer has become one of the leading causes of death in women worldwide [1]. Breast cancer not only causes serious physical harm to patients but also places a heavy burden on patients’ mental health and social functioning. Lymph node metastasis plays an important role in the diagnosis and prognosis of breast cancer [2]. Lymph nodes are important body tissue types that function to filter and remove waste products, bacteria and tumour cells from the body [3]. When breast cancer progresses to a certain stage, cancer cells have the potential to metastasise to lymph nodes through the lymphatic system, which is considered a marker of disease progression and metastasis [4]. Therefore, assessing the presence of lymph node metastasis in breast cancer patients is important for guiding treatment decisions and predicting patient prognosis.

Currently, assessment methods for lymph node metastasis in breast cancer include clinical, imaging and invasive examinations. Clinical examination mainly includes physical examination and lymph node palpation, which can provide preliminary diagnostic information [5]. Imaging examinations, such as ultrasound, computed tomography (CT), magnetic resonance imaging (MRI) and positron emission tomography (PET-CT) can provide more accurate information on lymph node metastasis [6]. Invasive tests, such as lymph node biopsy and lymphadenectomy, can obtain lymph node tissue samples directly that may help to confirm the diagnosis and staging [7]. However, although these assessment methods are widely used in breast cancer patients, they each have limitations. Clinical examination is limited by physician experience and palpation technique, and lymph nodes may not always be accurately judged for involvement. Imaging studies, while capable of providing detailed structural information, have limitations in detecting micronodal metastases or assessing the extent of metastases. Invasive tests, while providing definitive results, are somewhat limited by their aggressive nature and associated risks [8]. Therefore, to assess lymph node metastasis more accurately in breast cancer patients, it is of great clinical significance that new and more accurate non-invasive assessment methods be developed.

In recent years, the rapid development of artificial intelligence (AI) algorithms has brought new opportunities for the imaging diagnosis of breast cancer. Artificial intelligence technology is based on large-scale data training and deep learning algorithms, which can automatically extract features from medical images and perform accurate analysis and judgment [9]. In the diagnosis of breast cancer, artificial intelligence algorithms play an increasingly important role in the imaging field. First, AI algorithms can extract rich information from the image data of breast cancer patients and help doctors perform accurate assessments of tumour development and progression. Through deep learning and neural network technology, AI algorithms can automatically identify breast cancer-related lesion characteristics, such as the shape, size and edge characteristics of the mass, thereby helping doctors to quickly locate and diagnose the patient’s condition [10]. Second, AI algorithms can effectively solve the subjectivity and difference problems present in traditional imaging diagnoses. Because of the complexity of breast cancer imaging characteristics, physicians may interpret the same imaging result differently. Artificial intelligence algorithms, on the other hand, have high consistency and objectivity and can accurately automate judgment according to a large number of training data and algorithm models, reducing the diagnostic differences between doctors [11]. In addition, an AI algorithm has the advantages of processing large-scale data and rapid analysis, which can quickly process complex breast imaging data, reduce the work burden of doctors and improve diagnostic efficiency. Compared with the traditional manual reading method, AI algorithms can realise automatic image analysis and diagnosis, greatly shortening the diagnosis time and improving the early diagnosis rate and treatment effect of breast cancer [12]. Studies have shown that radiomics and AI can ‘see’ features that are generally invisible to the human eye in medical images. These new features have potential value in staging, prognosis and biological evaluation [13].

At present, although some studies have reported the application of AI algorithms in the evaluation of lymph node metastasis in breast cancer, a lack of up-to-date systematic evaluation to comprehensively evaluate the performance of AI algorithms alongside CT and MRI in terms of diagnostic accuracy remains. By performing a systematic review and meta-analysis, this study aims to analyse and summarise the data of existing studies on AI algorithm-assisted CT and MRI in the assessment of breast cancer lymph node metastasis and evaluate its diagnostic accuracy, sensitivity and specificity. This can help clinicians to better understand and apply AI algorithms in the evaluation of breast cancer lymph node metastasis, improve the accuracy of early diagnosis and the reliability of treatment decisions and ultimately improve the treatment outcome and survival rates of breast cancer patients.

## Methods

This study reports systematic reviews and meta-analyses of diagnostic test accuracy studies according to the preferred reporting items of the PRISMA-DTA guidelines [14].

### Search strategy and literature screening

We performed an extensive literature search to collect as much relevant research data as possible. We searched three electronic databases, PubMed, Embase and the Cochrane Central Register of Controlled Trials (CENTRAL), covering the time period from their inception to 18 June 2023. Medical Subject Headings (MeSH)/Emtree vocabulary was combined with free words, and keywords were set as the search mode for titles and abstracts. In addition, we manually searched the reference lists of relevant studies, reviews and meta-analyses for additional papers to ensure that no possible study articles were missed. Two researchers independently performed trial selection according to pre-specified inclusion criteria and imported the literature into Endnote X9.3.3 (Clarivate Analytics, London, UK) for management. Repeated or non-compliant studies were excluded. Eligible studies were identified by screening the titles, abstracts and full texts of all articles. Significant were extracted by two researchers using a pre-created data collection form. During data collection, if there were discrepancies between the two researchers, resolutions were discussed with the assistance of a third researcher.

The search strategy was as follows: (((‘Lymph Nodes’[Mesh]) OR (‘lymph’[Title/Abstract])) AND ((‘Breast Neoplasms’[Mesh]) OR (‘breast cancer’[Title/Abstract]))) AND (((((‘Magnetic Resonance Imaging’[Mesh]) OR (‘magnetic resonance imaging’[Title/Abstract])) OR (‘MRI’[Title/Abstract])) OR (((‘Tomography, X-Ray Computed’[Mesh]) OR (‘computed tomography’[Title/Abstract])) OR (‘CT’[Title/Abstract]))) AND (((‘Artificial Intelligence’[Mesh]) OR (‘Artificial Intelligence’[Title/Abstract])) OR (‘AI’[Title/Abstract]))).

### Inclusion and exclusion criteria

This study followed PICOS principles to ensure the reasonable control and comparison of five elements including study participants, interventions, controls, outcome measures and study design. Specifically, we included studies that met the following criteria: (1) breast cancer patients; (2) intervention vs. control based on an AI algorithm for CT or MRI imaging vs. pathologic diagnosis of lymph node metastasis (with pathology as the reference standard); (3) the primary outcome measure was the area under the receiver operating characteristic curve (AUC), and secondary outcomes included sensitivity, specificity, positive and negative likelihood ratios and diagnostic odds ratios; (4) the research design was cohort or case-control studies; and (5) language restriction was English. Concurrently, we excluded studies that met the following criteria: (1) duplicate studies with similar data; (2) unrelated study types such as animal studies, case reports, literature reviews or conference abstracts; and (3) studies with incomplete data or no reported set outcomes. By applying the above inclusion and exclusion criteria, we aimed to ensure the quality and reliability of the study and minimise potential deviations and errors.

### Data extraction and risk of bias assessment

Two investigators independently performed data extraction and a third resolved any discrepancies between them. From each included study, the following data were extracted: the first author’s surname, publication year, study design, sample size, lymph node metastasis definition, number of lesions, participant origin, ‘gold standard’ and diagnostic accuracy, specific algorithm model, instrumentation, use of clinical information (e.g. age, tumour stage, biomarker expression) and AUC results. The collected data were fourfold table data (2 × 2) including true positives (TPs), true negatives (TNs), false positives (FPs) and false negatives (FNs). Two researchers independently assessed the methodological quality using the Quality Assessment of Diagnostic Accuracy Studies (QUADAS-2) tool. The QUADAS-2 tool includes patient selection, index testing, reference standards, processes and timing. Disagreements between any two researchers were resolved by discussion or consultation with a third researcher.

### Statistical analysis

This study aimed to investigate the performance of AI-assisted CT and MRI imaging models in the diagnostic accuracy of lymph node metastasis in breast cancer patients by meta-analysis. RevMan 5.4 software and Stata SE 15.0 software were used for data analyses. Sensitivity and specificity were calculated based on FNs, FPs, TNs and TPs and graphically presented with boxes indicating values and horizontal lines indicating confidence intervals (CIs). The total receiver operating characteristic (SROC) curve was used to represent the performance of a diagnostic test. According to the AUC, rough classification accuracy guidelines were established as follows: 0.90–1 (excellent), 0.80–0.90 (good), 0.70–0.80 (fair), 0.60–0.70 (poor) and 0.50–0.60 (unqualified). Summary statistics and their 95% CIs were also calculated for the positive likelihood, negative likelihood and diagnostic odds ratios. Cochran’s Q test, combined with an I^2^ statistic, was used to assess the heterogeneity of the included study results. According to the degree of heterogeneity, a fixed effect or random effect model was used for meta-analysis. Funnel plots were employed to assess the potential for publication bias, and sensitivity analyses were used to assess the stability of the results. Clinical utility was assessed using Fagan plots, which provided the pretest probability of lymph node metastasis when calculating the post-test probability.

## Results

### Literature search

**Fig. 1:**
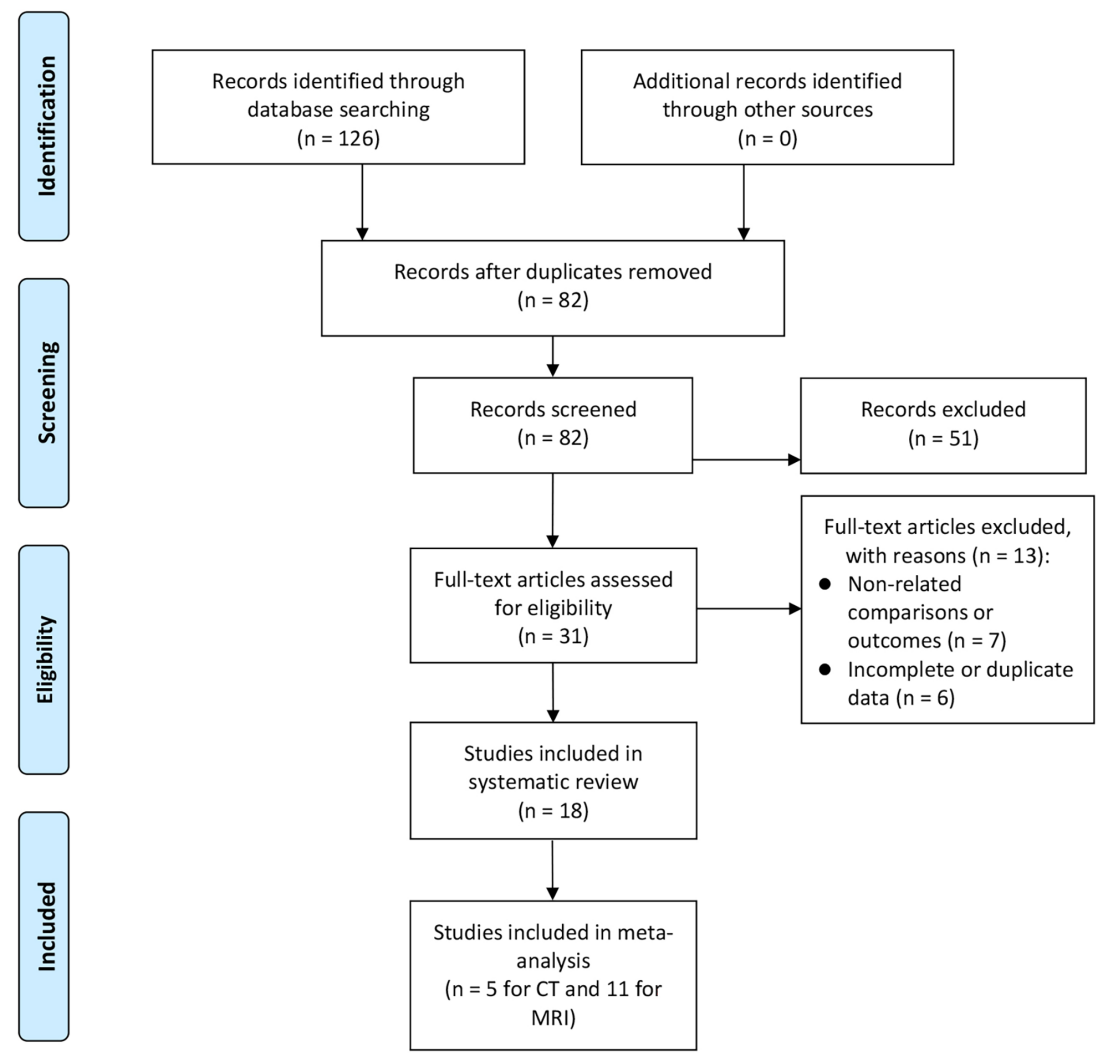
Study Inclusion Flow Chart

Figure 1 summarises the search and screening results for the relevant studies. Initially, we obtained 126 articles from the database search. By removing duplicate records manually and using software, we removed 44 duplicate articles. Subsequently, we removed 51 articles not related to the research topic by browsing the titles and abstracts and finally selected 31 for full-text reading. During full-text reading, we excluded 13 articles because their outcome measures, comparison strategies or incomplete data were not relevant to our study. Finally, we included 18 articles for systematic review and 16 articles involving 4,764 patients for meta-analysis [15–32].

### Basic characteristics of included literatures

The characteristics of each eligible study are detailed in Tables 1 and 2. Five studies targeted CT while 11 targeted MRI. Almost all studies used a retrospective design, with most being single-centre studies and only 2 being multi-centre types. In 16 eligible studies, different AI algorithms were adopted for modelling, 5 of which used clinical information in addition to images. In image-omics research, common machine learning classifiers include support vector machines, random forest methods and XGBoost. Of note, only 2 of the 16 studies used independent external validation methods, while others used internal validation methods, including cross-validation, randomly dividing datasets or distinguishing data in chronological order. Among the included studies, there were some differences in the definition of lymph node metastasis, with 14 studies exploring sentinel or axillary lymph node metastases in breast cancer patients, one study investigating the burden of axillary lymph node metastasis and another study considering residual lymph node metastasis. Nearly all studies identified lymph node metastases by pathological examination, including surgical resection or needle biopsy, while one study used 18FDG-PET for indirect assessment.

**Table 1:** Baseline data of included studies

Studies	Design	Data source	Country	Populations	Number of Patients	Age(years)	Validation method
AI-assisted CT							
Lee, 2022 [15]	Retrospective	Single center	Korea	Patients with clinically node-positive breast cancer treated with neoadjuvant chemotherapy	226	51.4 ± 9.3	Random splitting
Li, 2021 [16]	Retrospective	Single center	Japan	Patients with newly diagnosed invasive breast cancer	410	59.2 (28–90)	Cross-validation
Liu, 2021 [17]	Retrospective	Single center	China	Patients with breast cancer	401	N/A	Random splitting
Park, 2019 [18]	Prospective	Single center	Korea	Patients with invasive breast cancer	241	51 (25–84)	Random splitting
Song, 2021 [19]	Retrospective	Single center	Korea	Breast cancer patients without neoadjuvant chemotherapy	100	N/A	Random splitting
Yang, 2019 [20]	Retrospective	Single center	China	Patients with breast cancer	348	50 ± 10.66; 51.98 ± 9.43; 52.73 ± 9.84; 50.17 ± 9.68	Temporal validation
Zhang, 2022 [21]	Retrospective	Single center	China	Patients with non-specific invasive breast cancer	193	51.8 ± 8.7; 49.4 ± 9.9	Random splitting
AI-assisted MRI							
Arefan, 2020 [22]	Retrospective	Single center	USA	Patients newly diagnosed with invasive breast cancer	154	46.1 ± 10.3	Random splitting
Cui, 2019 [23]	Retrospective	Single center	China	Patients with benign primary breast carcinoma	102	Range: 35–60	Cross-validation
Fusco, 2018 [24]	Retrospective	Single center	Italy	Patients newly diagnosed with invasive breast cancer	52	Range: 31–58	Cross-validation
Han, 2019 [25]	Retrospective	Single center	China	Patients with breast cancer	411	52.10 ± 9.7 (Training); 51.95 ± 10.4 (Validation)	Temporal validation
Liu, 2019 [26]	Retrospective	Single center	China	Patients with histologically confirmed breast cancer	62	48.14 ± 8.35; 49.78 ± 12.53	Random splitting
Luo, 2018 [27]	Retrospective	Single center	China	Patients confirmed to breast cancer by histological diagnosis	172	N/A	Random splitting
Ren, 2020 [28]	Retrospective	Single center	USA	Patients who had unilateral breast cancer and were treated with neoadjuvant chemotherapy	99	N/A	Cross-validation
Tan, 2020 [29]	Retrospective	Single center	China	Patients initially diagnosed as invasive breast carcinoma	329	48.94 ± 10.97 (Training); 47.03 ± 8.14 (Validation)	Temporal validation
Yu, 2021 [30]	Retrospective	Multicenter	China	Patients with early-stage invasive breast cancer	1088	N/A	External validation
Zhang, 2019 [31]	Retrospective	Single center	China	Patients with breast cancer	146	46.70 ± 11.85 (Training); 47.32 ± 9.18 (Validation)	Random splitting
Zhang, 2021 [32]	Retrospective	Multicenter	China	Patients who had early-stage invasive breast cancer	230	51.38 ± 11.67 (Training); 50.45 ± 9.90 (Validation); 47.52 ± 11.90 (Test)	External validation

AI: artificial intelligence; CT: computed tomography; MRI: magnetic resonance imaging. Age was expressed as mean ± standard deviation or median (range)

**Table 2:** Basic characteristics of CT or MRI prediction models based on artificial intelligence algorithms

Studies	AI algorithm	Equipment	Clinical information	Reference Standard	Outcome Definitions	AUC
AI-assisted CT						
Lee, 2022 [15]	Boruta, gradient-boosting classifier	Siemens and GE	Yes	Surgical resection	Residual ALN metastasis	0.866
Li, 2021 [16]	DCNNs	^18^FDG-PET/CT (Philips and GE)	No	Surgical resection	ALN metastasis	0.868
Liu, 2021 [17]	DA-VGG19	GE and Philips	No	Surgical resection	ALN metastasis	0.9694
Park, 2019 [18]	DT, RF, NB, SVM, ANN	Philips	No	Surgical resection	ALN metastasis	0.86
Song, 2021 [19]	XGBoost	^18^FDG-PET/CT (GE)	No	Surgical resection	ALN metastasis	0.89
Yang, 2019 [20]	CNN-fast	GE and Philips	No	Surgical resection	SLN metastasis	0.817
Zhang, 2022 [21]	Lasso regression	Philips	Yes	Surgical resection	SLN metastasis	0.95
AI-assisted MRI						
Arefan, 2020 [22]	LDA, RF, NB, KNN, SVM	3.0 T Siemens	No	FNA or surgical resection	ALN metastasis	0.82
Cui, 2019 [23]	SVM, KNN, LDA	3.0 T Siemens	No	FNA or surgical resection	ALN metastasis	0.8615
Fusco, 2018 [24]	LDA	1.5 T Aurora	No	Surgical resection	ALN metastasis	0.812
Han, 2019 [25]	SVM	1.5 T GE	Yes	Surgical resection	ALN metastasis	0.87
Liu, 2019 [26]	SVM, XGBoost	3.0 T GE	No	Surgical resection	ALN metastasis	0.83
Luo, 2018 [27]	SVM	1.5 T Philips	No	Surgical resection	SLN metastasis	0.852
Ren, 2020 [28]	CNN	1.5 T GE	No	^18^FDG-PET	ALN metastasis	0.91
Tan, 2020 [29]	SVM	3.0 T GE	Yes	Surgical resection	ALN metastasis	0.810
Yu, 2021 [30]	RF	N/A	Yes	Surgical resection	ALN metastasis	0.91
Zhang, 2019 [31]	RF	1.5 T Philips	No	Surgical resection	SLN metastasis	0.868
Zhang, 2021 [32]	Lasso regression	1.5 T Siemens	No	Surgical resection	ALN metastatic burden	0.81

AI: artificial intelligence; ALN: axillary lymph node; ANN: artificial neural network; AUC: area under the curve; CNN: convolutional neural network; CT: computed tomography; DA: deformable attention; DCNNs: deep convolutional neural networks; DT: decision tree; FNA: fine-needle aspiration; GA: genetic algorithm; MRI: magnetic resonance imaging; NB: naïve Bayes; RF: random forest; SLN: sentinel lymph node; SVM: support vector machine; ^18^FDG-PET: fluorodeoxyglucose positron emission tomography

### Risk of bias assessment

**Fig. 2:**
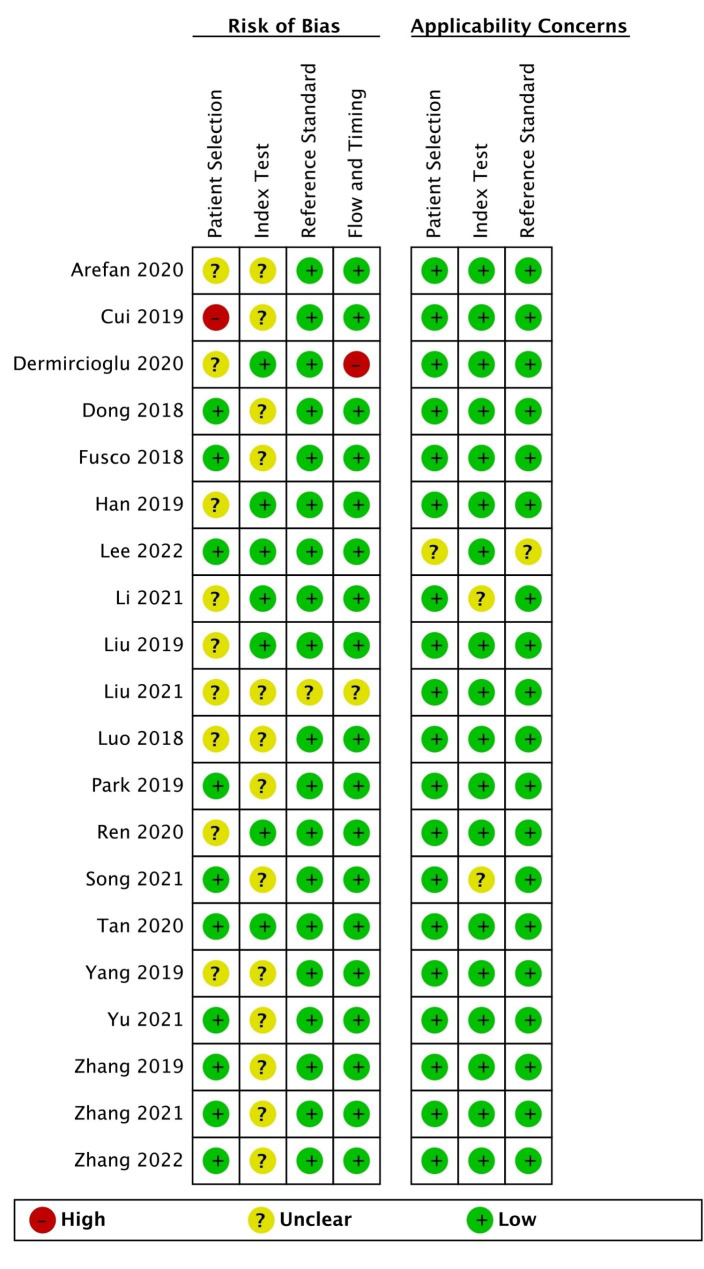
Methodological quality assessment of included literatures

The methodological quality of the 16 included studies is shown in Fig. 2. Because the narratives were unclear, 9 studies had an unclear risk of bias in the field of ‘patient selection’ and 1 indicated a high risk; 13 studies had an unclear risk of bias in the field of ‘index test’ because a blinded setting was not accounted for. Only 1 study had an unclear risk of bias score in the field of ‘reference standard’ because the mode of pathological examination was not described in detail. It is important to note that 3 studies may have included high levels of concern regarding patient selection and the ‘index test’ aspect, given their degree of agreement with the questions of this review.

### Meta-analysis

**Fig. 3:**
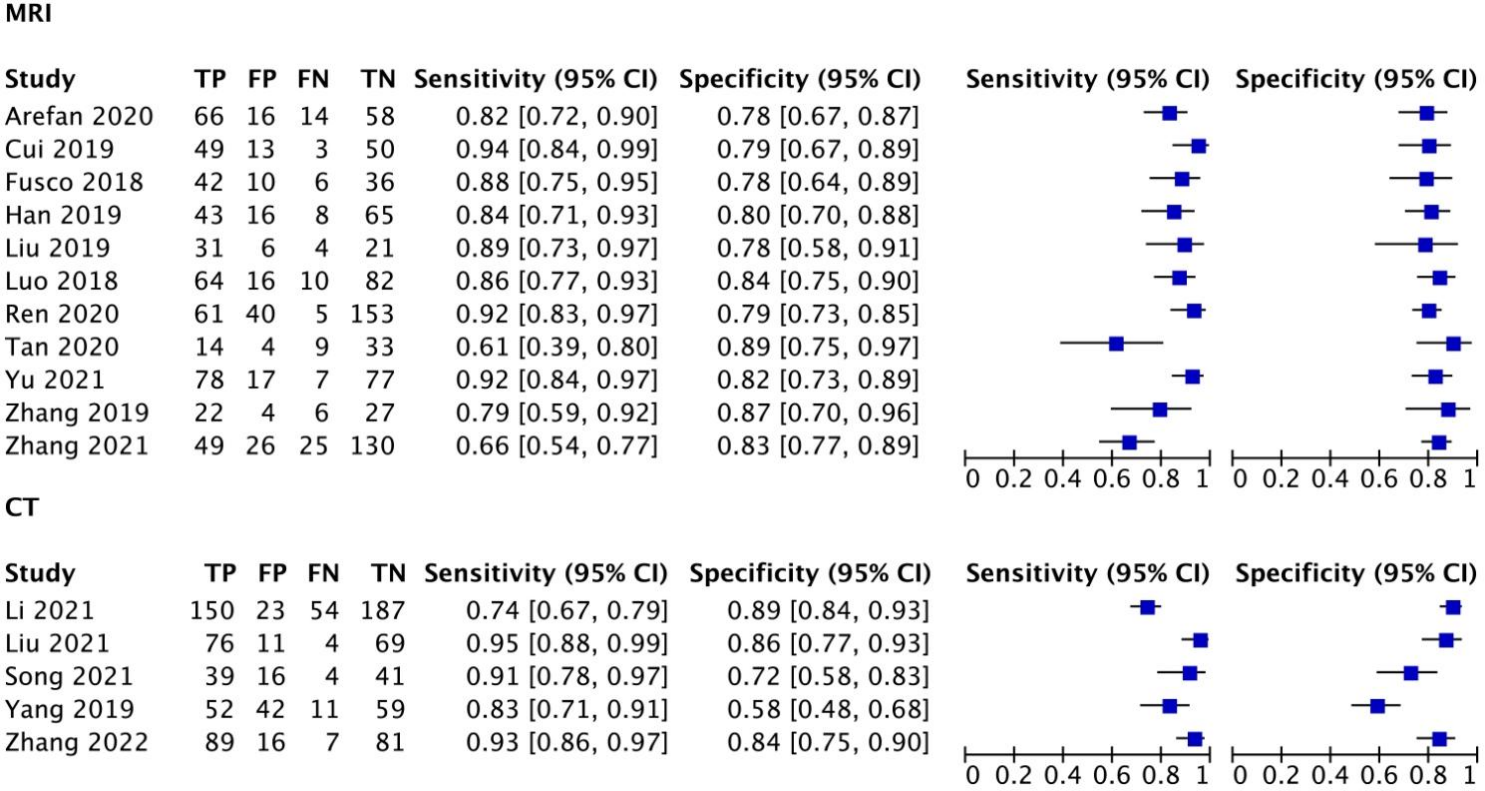
Forest map of prediction of lymph node metastasis in breast cancer patients by CT or magnetic resonance based on artificial intelligence algorithm

**Fig. 4:**
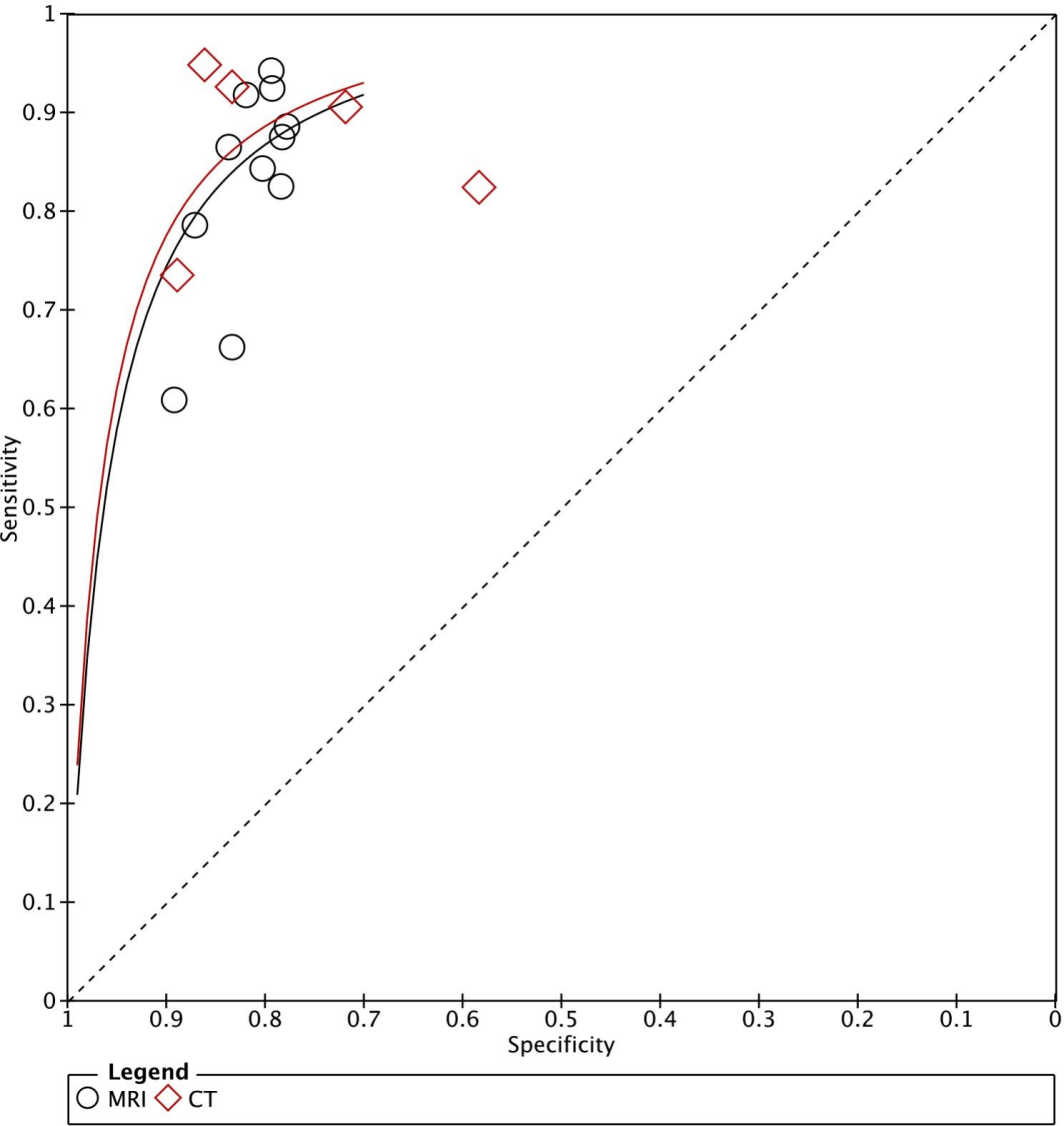
SROC Plot of CT or MRI Based on Artificial Intelligence Algorithm for Predicting Lymph Node Metastasis in Breast Cancer Patients

The results of a meta-analysis study using AI for lymph node metastasis risk calculation in breast cancer patients are shown in Fig. 3. As indicated, 11 studies used a manual algorithm MRI to calculate breast cancer risk, which had sensitivity of 0.85 (95% CI 0.79–0.90; *p* < 0.001; I^2^ = 75.3%), specificity of 0.81 (95% CI 0.66–0.83; *p* < 0.001; I^2^ = 0%), a positive likelihood ratio of 4.6 (95% CI 4.0–4.8), a negative likelihood ratio of 0.18 (95% CI 0.13–0.26) and a diagnostic odds ratio of 25 (95% CI 17–38). Five studies used a manual algorithm CT to calculate breast cancer risk, which had sensitivity of 0.88 (95% CI 0.79–0.94; *p* < 0.001; I^2^ = 87.0%), specificity of 0.80 (95% CI 0.69–0.88; *p* < 0.001; I^2^ = 91.8%), a positive likelihood ratio of 4.4 (95% CI 2.7–7.0), a negative likelihood ratio of 0.15 (95% CI 0.08–0.27) and a diagnostic odds ratio of 30 (95% CI 12–72). In addition, we also used SROC plots to represent the results of a meta-analysis study, based on the use of AI algorithms, to assist MRI or CT in calculating the risk of lymph node metastases in breast cancer patients. As shown in Fig. 4, for MRI or CT, the AUC following the study summary was 0.85 (95% CI 0.82–0.88) and 0.91 (95% CI 0.88–0.93), respectively. These results suggest that the predictive ability of AI algorithms to analyse MRI and CT images for the risk of lymph node metastases in breast cancer patients was classified as good and excellent.

### Publication bias and sensitivity analysis

**Fig. 5:**
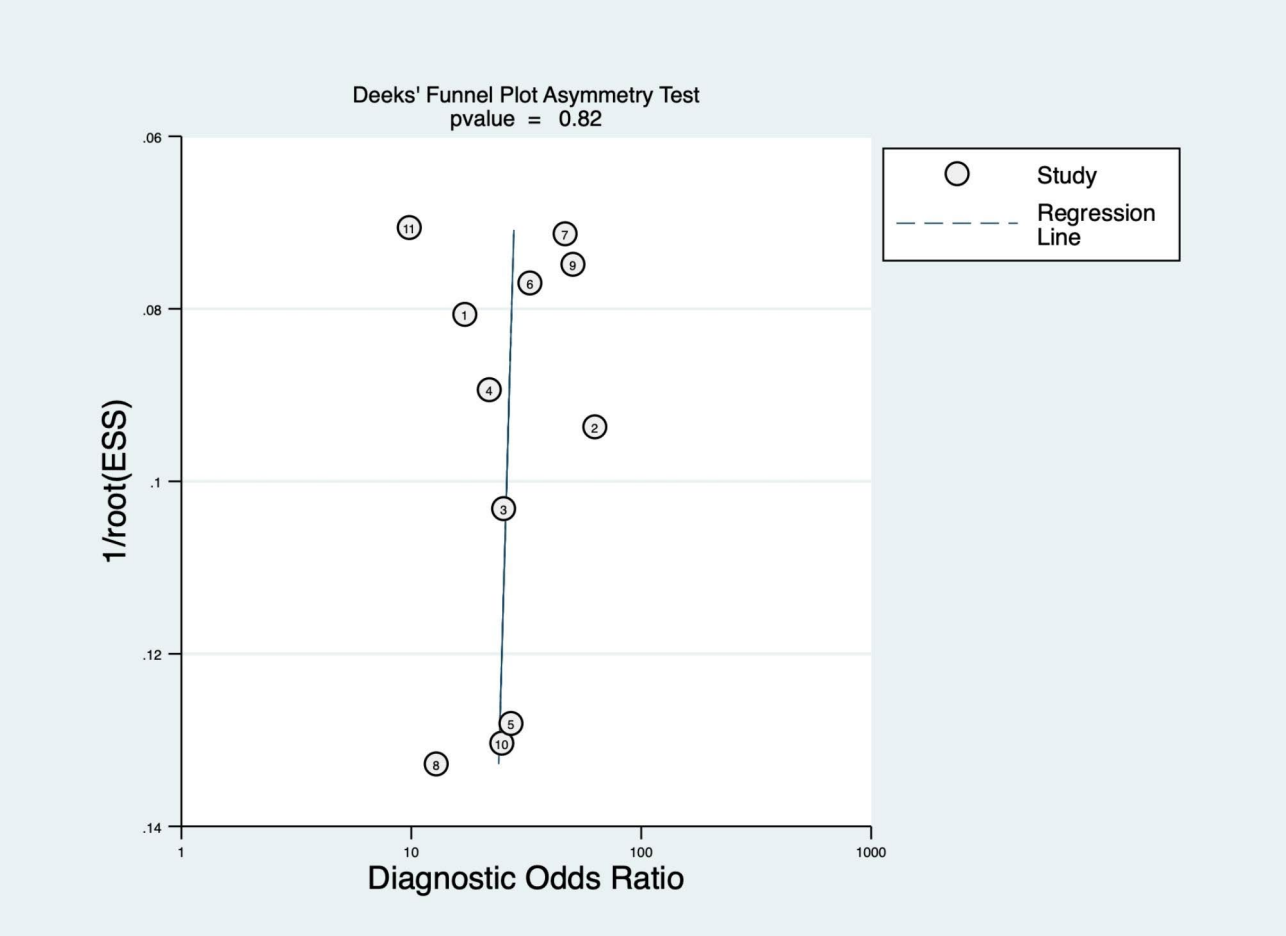
Funnel plot of MRI based on artificial intelligence algorithm for predicting lymph node metastasis in breast cancer patients

**Fig. 6:**
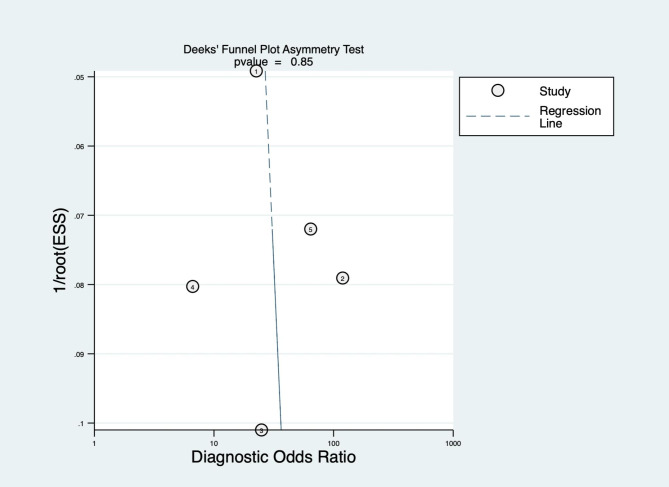
Funnel plot of CT based on artificial intelligence algorithm for predicting lymph node metastasis in breast cancer patients

We performed a publication bias analysis of the included studies, as shown in Figs. 5 and 6, and the funnel plot asymmetry test showed no significant publication bias for the included MRI and CT studies (*p* = 0.82 and 0.85, respectively). While performing the meta-analysis, we also performed a sensitivity analysis. After each exclusion of a single study, there was no large variation in the results, suggesting the stability of the findings; however, heterogeneity between studies remained significant.

### Clinical utility

**Fig. 7:**
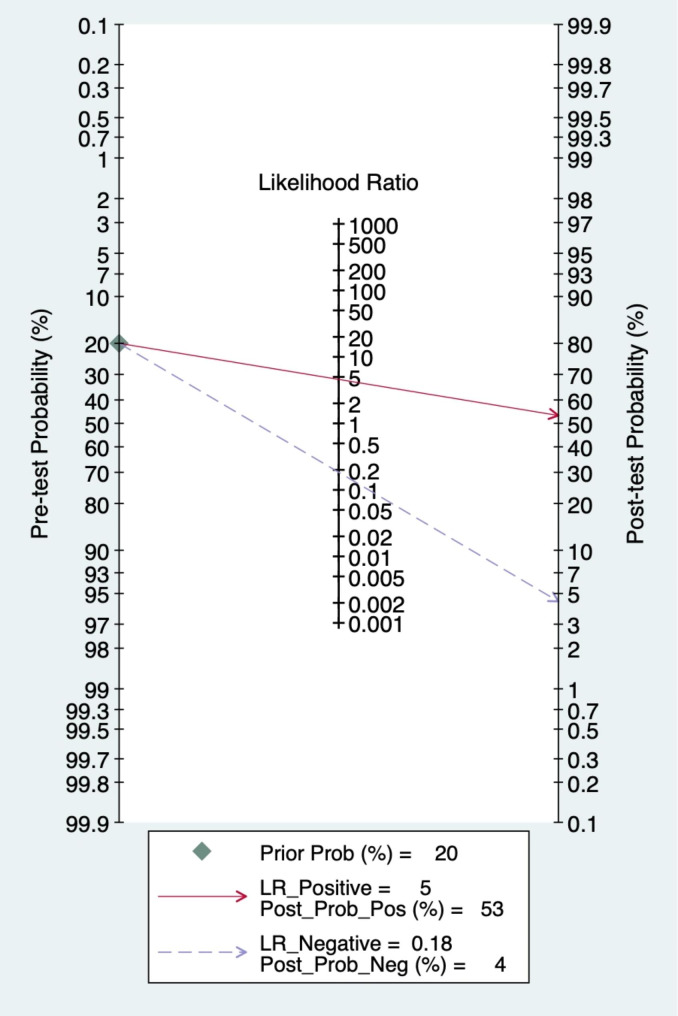
Fagan plot of MRI based on artificial intelligence algorithm for predicting lymph node metastasis in breast cancer patients

**Fig. 8:**
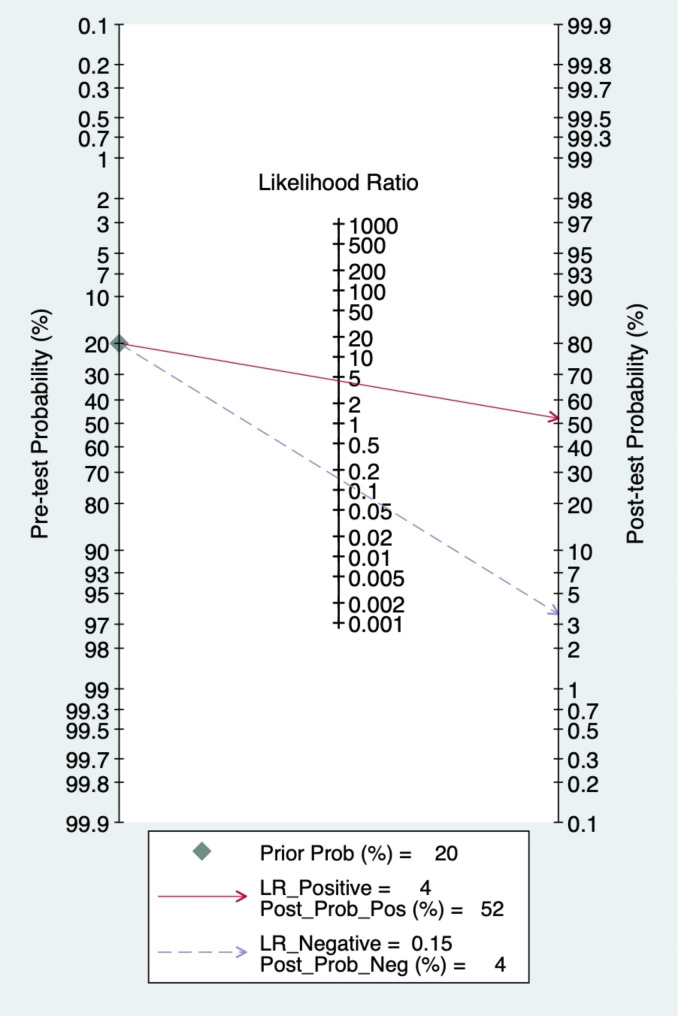
Fagan Plot of CT Predicting Lymph Node Metastasis in Breast Cancer Patients Based on Artificial Intelligence Algorithm

Using an AI-based radiomics MRI model, the post-test probability was increased from 20 to 53% with a positive likelihood ratio of 5 when the pre-test was positive and decreased to 4% with a negative likelihood ratio of 0.18 when the pre-test was negative (Fig. 7). Using the AI-based radiomics CT model, the post-test probability was increased from 20 to 52% with a positive likelihood ratio of 4 when the pretest was positive and a decrease to 4% with a negative likelihood ratio of 0.15 when the pre-test was negative (Fig. 8).

## Discussion

The main work of this study comprises a meta-analysis of ultrasound images for predicting lymph node metastasis in breast cancer patients based on AI algorithms. We comprehensively analysed several recent studies and evaluated the application effect of an AI algorithm in this field. The results showed that MRI and CT images based on an AI algorithm showed high predictive accuracy and reliability in the diagnosis of lymph node metastases in breast cancer patients. This is essential for physicians to guide individualised treatment planning, selecting appropriate treatment strategies and reducing the risk of misdiagnosis and missed diagnosis. In addition, imaging analysis using AI algorithms helps to reduce unnecessary needle biopsies and surgical resection, thereby reducing the physical and mental burden on patients and improving treatment outcomes and quality of life. These findings provide important support and guidance for clinical practice and serve as a powerful tool for treatment decision-making and prognostic evaluation of breast cancer patients. Future studies and applications should further explore and optimise the application of AI algorithms in breast cancer diagnosis and treatment to enhance their clinical application value and potential.

An AI algorithm has multiple advantages over traditional examination methods in the evaluation of breast cancer lymph node metastasis. First, AI algorithms can automatically extract features from breast cancer CT and MRI images and perform highly accurate analysis and assessment. Through deep learning and neural network techniques, AI algorithms can identify and localise lymph node metastases in breast cancer, provide more reliable quantitative information and help physicians make accurate diagnostic decisions [33]. Second, an AI algorithm has advantages in the consistency of image interpretation. The image interpretation of breast cancer lymph node metastasis may reflect subjectivity and variation compared with the high consistency and objectivity of an AI algorithm. Through the training of large-scale data and the optimisation of algorithm models, AI algorithms can provide consistent and reliable diagnostic results, reduce diagnostic differences between doctors and improve the diagnostic consistency of breast cancer lymph node metastases [34]. In addition, an AI algorithm can also accelerate the image interpretation speed of breast cancer lymph node metastasis and improve work efficiency. Compared with the traditional manual interpretation method, AI algorithms can automatically analyse and interpret a large number of breast cancer image data, greatly shortening the diagnosis time. This means greater productivity and more timely diagnostic results for clinicians, providing patients with faster and more precise treatment decisions [35]. However, it should be noted that despite the many advantages of AI algorithms in the assessment of breast cancer lymph node metastases, there remain challenges and limitations [36, 37]. For example, training AI algorithms requires a large amount of high-quality labelling data, and the performance of algorithms may be affected by data bias and imbalance. In addition, the application of AI algorithms requires strict validation and regulation to ensure their accuracy, reliability and safety in clinical practice.

In conclusion, AI algorithms have shown great potential in the evaluation of breast cancer lymph node metastasis. Its advantages, such as accuracy, consistency and high efficiency, are expected to improve the accuracy of early diagnosis and treatment decisions for breast cancer lymph node metastasis and ultimately improve the prognosis of patients. However, further research and validation are needed to ensure the reliability and validity of AI algorithms in clinical practice and provide better medical services for patients.

This meta-analysis is the first to update the diagnostic value of MRI images in predicting lymph node metastasis in breast cancer patients based on AI algorithms interpreting CT images. In the past, studies explored the use of imaging in breast cancer lymph node metastasis but few systematically integrated and evaluated this area. Existing studies focused on the assessment of breast cancer lymph node metastasis in MRI. As a high-resolution imaging technique, MRI has good soft tissue contrast and spatial resolution and is widely used in the diagnosis and evaluation of breast cancer. Studies analysed lymph node metastases in breast cancer patients by MRI and achieved specific research results [30, 38]. Zhang et al. [39] synthesised 13 articles and concluded that machine-learning-based MRI imageomics has the potential to accurately predict axillary and sentinel lymph node metastasis, with pooled sensitivity, specificity and AUC reaching 0.82, 0.83 and 0.89, respectively. However, Chen et al. [40] found that MRI sequences and algorithms were the main factors affecting the diagnostic accuracy in machine learning-assisted MRI for the judgment of axillary lymph node metastasis. Despite its good sensitivity and negative predictive value, machine learning-assisted MRI still overlooked 20% of patients with axillary lymph node metastases, an undoubtedly fatal result for patients, indicating that it remains non-applicable in daily diagnosis and treatment. Further exploration is needed in the future to improve the accuracy and practical efficiency of AI algorithms.

Compared with conventional mammography and ultrasonography, CT and MRI have unique advantages and differences in the assessment of breast cancer lymph node metastasis. First, CT and MRI have higher resolution and clearer image quality in anatomical structure presentation and can show the location, size and morphological characteristics of lymph nodes. Computed tomography provides high-resolution cross-sectional images, whereas MRI provides more detailed tissue contrast through multiple sequences and contrast enhancement techniques. These characteristics contribute to the qualitative and quantitative analysis of lymph node metastasis [41]. Second, CT and MRI can provide more functional information; CT can be combined with an intravenous contrast agent for lymph node staging and the assessment of its blood supply and haemodynamic characteristics, while MRI can obtain metabolic information about breast lesions through different sequences, such as dynamic contrast-enhanced MRI and magnetic resonance spectroscopy. This functional information can help to assess the activity of the lesion and predict the prognosis of the patient [42]. However, CT and MRI also include limitations and considerations relative to mammography and ultrasonography. First, CT and MRI require patients to enter a device for examination, which is relatively time-consuming and costly. The MRI approach requires a high degree of cooperation from patients and presents limitations for those with, for example, metallic implants or cardiac pacemakers. In contrast, mammography and ultrasonography are more convenient, economical and suitable for most breast cancer patients, especially for women with higher breast density and pregnant women [43]. As previously stated, CT and MRI have higher resolution, clearer image quality and provide more functional information than conventional mammography and ultrasonography in the assessment of breast cancer lymph node metastasis, but the selection of appropriate imaging tools requires comprehensive consideration of patient characteristics, clinical needs feasibility and decision-making.

This study includes some limitations that impacted the interpretation and generalisability of the results. First, the number and quality of studies available for meta-analysis was limited. Although AI algorithms have received much attention related to predicting breast cancer lymph node metastasis, there may still be relatively few alternative studies. This may be because the application of AI algorithms is still in the development stage and related research remains ongoing. In addition, there may be differences between studies, including variations in study design, sample size, data collection and assessment methods, leading to heterogeneity of the results. We included only English literature in the present study, which may have contributed to a language bias. Relevant studies in other languages may not have been included in the analysis, which could have impacted our results. Reporting algorithm performance and the selection of assessment metrics may also differ. Different studies may use different evaluation indicators and thresholds to evaluate the performance of AI algorithms. This variability may lead to heterogeneity in the results and inconsistency in comparisons. In addition, potential data pooling issues may have been present due to heterogeneity of the data and differences in standardisation methods. Furthermore, different data sources, acquisition methods, image quality and feature extraction methods may have been applied in the different studies derived from the literature search. In addition, the predictive performance of the algorithm may vary for different types of breast cancer lymph node metastasis (such as micrometastasis and giant metastasis), which must also be carefully considered.

## Conclusion

In summary, the results of this study showed that CT and MRI based on AI algorithm analysis yielded similar and better diagnostic accuracy in predicting lymph node metastasis in breast cancer patients. This indicated that these two imaging modalities have potential clinical application value and provide new ideas and methods for the diagnosis and treatment of breast cancer. Further studies are still needed to validate the results of this study and promote the clinical application of AI and imaging in this field in the future.

## Data Availability

The datasets used and analyzed during the current study are available from the corresponding author on reasonable request.
